# Tunable optical spin Hall effect in a liquid crystal microcavity

**DOI:** 10.1038/s41377-018-0076-z

**Published:** 2018-10-10

**Authors:** Katarzyna Lekenta, Mateusz Król, Rafał Mirek, Karolina Łempicka, Daniel Stephan, Rafał Mazur, Przemysław Morawiak, Przemysław Kula, Wiktor Piecek, Pavlos G. Lagoudakis, Barbara Piętka, Jacek Szczytko

**Affiliations:** 1 Institute of Experimental Physics, Faculty of Physics, University of Warsaw, Warsaw, Poland; 2 Institute of Applied Physics, Military University of Technology, Warsaw, Poland; 3 Institute of Chemistry, Military University of Technology, Warsaw, Poland; 4 Department of Physics and Astronomy, University of Southampton, Southampton, SO17 1BJ UK; 5 Skolkovo Institute of Science and Technology Novaya St.,100, Skolkovo, 143025 Russian Federation

## Abstract

The spin Hall effect, a key enabler in the field of spintronics, underlies the capability to control spin currents over macroscopic distances. The effect was initially predicted by D'Yakonov and Perel^1^ and has been recently brought to the foreground by its realization in paramagnetic metals by Hirsch^2^ and in semiconductors^3^ by Sih et al. Whereas the rapid dephasing of electrons poses severe limitations to the manipulation of macroscopic spin currents, the concept of replacing fermionic charges with neutral bosons such as photons in stratified media has brought some tangible advances in terms of comparatively lossless propagation and ease of detection^4–7^. These advances have led to several manifestations of the spin Hall effect with light, ranging from semiconductor microcavities^8,9^ to metasurfaces^10^. To date the observations have been limited to built-in effective magnetic fields that underpin the formation of spatial spin currents. Here we demonstrate external control of spin currents by modulating the splitting between transverse electric and magnetic fields in liquid crystals integrated in microcavities.

## Introduction

The conversion between angular and spin momenta of light can be treated as the analogue of spin-orbit coupling or the motion of charged particles in magnetic fields. Such conversion in optics is related to the optical spin Hall effect^4^ (OSHE): the “magnitude” of the artificial “magnetic field” acting on a photon is directly related to TE/TM splitting of photonic modes in a medium. In this paper, we propose to exploit OSHE, initially predicted in microcavities by Kavokin et al.^4^, to control the spin and momentum states of polaritons, i.e., strongly interacting light and matter. Nonetheless, the situation is significantly different due to a matter component. Previous studies have shown the OSHE in both the strong coupling regime^8^ and a bare cavity^9^. Photons populating a Rayleigh ring experience different phase shifts; this results in the appearance of different spin patterns of light. However, tunability is absent in all of the above structures. We propose to tune TE/TM splitting by rotation of the molecular director of birefringent liquid crystal filling our cavity by application of an external voltage. We demonstrate the use of an external electric field to modify a light polarization pattern, causing anisotropic transmission through the cavity and leading to a much broader range of polarization textures than normally observed in optical cavities.

**Fig. 1:**
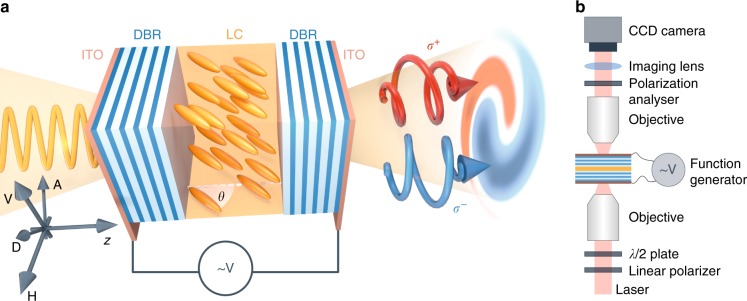
Sample and experimental setup. **a** Schematic structure of the tunable cavity in transmission configuration where the degree of the circular polarization can be measured. Sample substrate consists of an indium tin oxide transparent electrode (ITO) and two dielectric mirrors (DBR). The cavity between the parallel DBRs is filled with LC. Using external electric field $\bar E$, the tilt angle *θ* of the liquid crystal molecular director and consequently the effective refractive index for the light at normal incidence can be changed. The experiment was performed for different polarizations of the incident light: vertical (V), horizontal (H), diagonal (D), and anti-diagonal (A). **b** Scheme of the experimental setup for transmission measurements

The uniqueness of our work is the development of a new type of microcavity (Fig. 1a). The prepared sample consists of two dielectric mirrors formed by a stack of several low/high refractive index SiO_2_/TiO_2_ layers with a 1.7 μm cavity between them filled with a nematic liquid crystal (LC). This structure is surrounded by transparent electrodes made of indium tin oxide (ITO). The energy position of the photon modes depends directly on the optical thickness of the cavity, the product of its refractive index and thickness. Here, the optically uniaxial liquid crystalline nematic structure (cavity material) is subjected to an electric field; hence, the effective refractive index can be controlled to tune the cavity modes. The birefringent nematic LC structure in the cavity exhibits an anisotropy of electric permittivity described with the tensor $\hat \varepsilon$, which is depicted with a uniaxial ellipsoid of revolution with the axis usually collinear with the molecular director and optical axis of the medium. The nematogenic mixture filling the cavity is a dual frequency liquid crystal^11^, which means that the material exhibits a positive dielectric permittivity anisotropy, Δ*ε* > 0, at low frequencies and a negative one, Δ*ε* < 0, at high frequencies (of the order of tens of kHz). This allows the driving of the optical axis of the medium toward the direction of the external electric field $\bar E$ at low frequencies and towards the plane perpendicular to it at high frequencies at voltages higher than a threshold voltage (here 8.2 V). Measurements were conducted in the transmission setup in k-space (imaging lenses) shown in Fig. 1b. The excitation and detection systems consist of a quarter wave plate, half wave plate and a linear polarizer, allowing for sensitive polarization measurements.

**Fig. 2:**
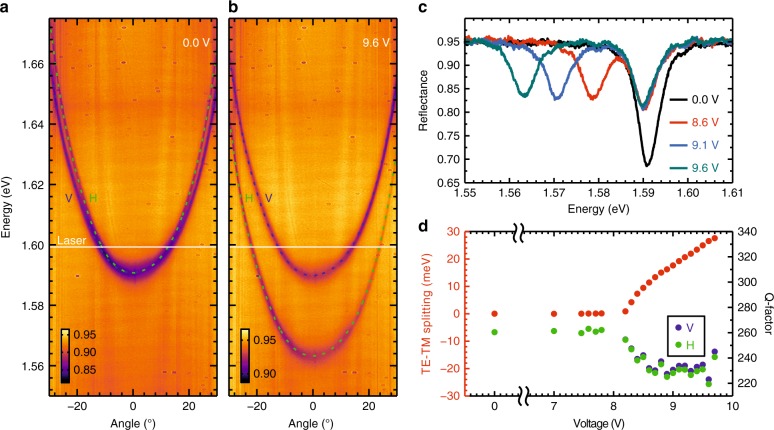
Tunability of liquid crystal microcavity. Applying voltage to the structure results in rotation of the liquid crystal molecules, which leads to controllable splitting of the cavity modes. Angle-resolved reflectance spectra **a** without and **b** with applied voltage. Positions of the cavity modes polarized in the vertical (V) and horizontal (H) directions are marked with green and purple dashed lines, respectively. The laser energy used in experiments is denoted with a white solid line. **c** Cross-sections of reflectance maps for normal incidence for four applied voltages showing tunability. **d** Dependence on the applied voltage of the energy splitting between horizontally and vertically polarized cavity modes for normal incidence (left axis) and Q-factor of horizontally and vertically polarized cavity modes (right axis)

In the absence of an applied voltage, the molecular director is aligned parallel to the growth axis that results in zero TE/TM splitting at normal incidence. In Fig. 2a, we show the cavity dispersion at zero voltage using white light reflectivity in *σ*^+^ circular polarization. The two distinctive modes (TE/TM) correspond to the two linear polarizations—horizontal/vertical. By applying an external voltage, we control the tilting of the molecular director in the plane perpendicular to the cavity plane, changing the effective refractive index in the direction of the tilt. The cavity is oriented so that the tilt of the molecules occurs in the vertical (V) polarization plane of incident light. In Fig. 2b, we show the dispersion of the cavity at 9.6 V. As expected, the vertical polarization mode remains virtually unchanged, while the horizontal polarization mode redshifts with respect to the zero-voltage case. Evidently, we can induce a TE/TM splitting by applying an external voltage. Figure 2c shows reflectivity spectra at normal incidence for several values of the voltage. For zero voltage, due to the zero TE/TM splitting, only one reflectivity minimum is observed (black line). For voltage values above 8.2 V, a splitting is observable, as evidenced by the two reflectivity minima. Figure 2d shows the measured TE/TM splitting (left axis) versus the amplitude of the applied voltage. The splitting is tunable between 0 and 27.6 meV. In the presence of the electric field, we observe that the linewidth of the reflectivity spectra broadens, resulting in ~10% reduction of the Q-factor ($\Delta \lambda /\lambda$). On the right axis of Fig. 2d, we plot the measured Q-factor for each value of the applied voltage separately for horizontal and vertical polarizations. The apparent reduction in the Q-factor is conceivably due to increased intracavity scattering in the presence of the field.

**Fig. 3:**
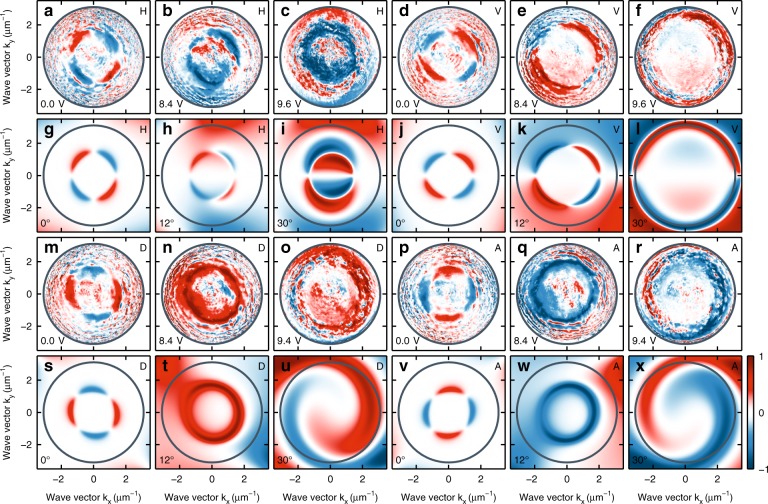
Tunable optical spin Hall effect in liquid crystal microcavity. Degree of circular polarization ρ_C_ for different voltages applied to the structure: **a**–**f**, **m**–**r** measurement, **g**–**l**, **s**–**x** model. The two upper panels correspond to the degree of circular polarization ρ_C_ for **a**–**c**, **g**–**i** horizontal (H) and **d**–**f**, **j**–**l** vertical (V) polarization of incident light. The next two panels correspond to the degree of circular polarization ρ_C_ for **m**–**o**, **s**–**u** diagonal (D) and **p**–**r**, **v**–**x** anti-diagonal (A) polarization of incident light. Circles mark the area available experimentally due to the numerical aperture of the objectives

Fundamental to the physics of the OSHE, the TE/TM splitting acts as a directionally dependent effective magnetic field that lies in the plane of the cavity. In the presence of TE/TM splitting, the rotation of the polarization vector depends on the direction of the in-plane propagation of the optical field (direction of in-plane wave vector)^4^. To map this dependence, we performed reciprocal space imaging of the degree of circular polarization (DCP) in the transmission configuration of a tightly focused (± 3.1 μm^−1^) monochromatic (1.599 eV) laser source for horizontal (H), vertical (V), diagonal (D), and anti-diagonal (A) linear polarizations of the laser. Figure 3 shows the experimental and modeled reciprocal space images of the DCP for three values of the externally applied voltage (0, 8.4, and 9.6 V). The first row (a–f) shows the DCP for horizontal (ac) and vertical (d–f) polarization. For 0 V and thus zero TE/TM splitting, we observe a quadrupole of the DCP that has opposite sign for horizontal (Fig. 3a) and vertical (Fig. 3d) polarization of the incoming beam. In the reciprocal images, the intensity of the transmitted beam is strongest within the transmission cone of the cavity. Scattering of light within the cavity and from mirror defects produces an unavoidable non-zero intensity distribution across the reciprocal image that leads to a non-zero DCP away from the transmission cone. Such observations were previously reported in semiconductor microcavities both in the strong and weak coupling regime^8,9^ and confirm the presence of the OSHE in LC filled cavities.

Unlike the case for epitaxial semiconductor microcavities, in LC filled cavities we can tune the TE/TM splitting by applying an external voltage. Figure 3b, c, e, f shows the DCP for horizontal (vertical) polarization of the incoming beam for an external voltage of 8.4 V (Fig. 3b, e) and 9.6 V (Fig. 3c, f), respectively. Evidently, the polarization pattern is strongly modulated as expected from the increasing TE/TM splitting. Furthermore, with increasing voltage we observe that the polarization pattern of higher DCP is enlarged in reciprocal space due to the redshift and consequent increase of the radius of the transmitted light cone for the horizontal polarization component. Figure 3m–o, p–r shows the DCP for diagonal (anti-diagonal) polarization of the laser. For zero applied voltage, we observe a 45° rotation of the quadrupole pattern of the DCP with respect to that observed for the horizontal (vertical) polarizations of the laser, as it was previously observed in the real-space DCP of an expanding polariton condensate in inorganic microcavities^12^. In the presence of an externally applied field, we observe a much richer texture of the DCP in reciprocal space.

To understand the extent to which the observed patterns originate from the OSHE or intracavity-disorder mediated polarization-dependent light scattering, we modeled the polarization state of light transmitted through our system. We used the Berreman method^13,14^, which takes into account the birefringence of the cavity medium. The rotation of the dielectric tensor of the liquid crystal layer inside the cavity was taken proportional to the applied voltage. Figure 3g–l, s–x shows the modeled DCP patterns for the different linear polarizations of the laser. Details of the model are described in the Methods section. Overall, we observed a good agreement between our measurements and the modeled patterns, taking into consideration the soft matter constituents of our intracavity layer. A broad spectrum of polarization patterns are realizable, ranging from those previously observed in epitaxial inorganic microcavities (see first and fourth columns of Fig. 3) to strongly asymmetric patterns such as those observed for diagonal/anti-diagonal polarization in the presence of a field. We note that the latter bears similarities with previously observed polariton spin whirls as shown in Fig. 3o, r, u, x)^15^.

In summary, we propose an active polarization converter that operates at room temperature and explores the extremely wide range of TE/TM splitting, not accessible in semiconductor microcavities. Moreover, the splitting is tunable by an external voltage. The idea is based on a photonic cavity with an effective wave retardation in an anisotropic LC cavity. Our approach can be easily extended to VIS-IR light and the telecom range using the same technology and the same materials. We demonstrated an OSHE in a new kind of microcavity, the construction of which allows for a direct introduction of any type of light-active particles inside, such as optical dyes^16,17^, quantum dots^18^, plasmonic particles^19^, or monolayers of transition metal dichalcogenides^20,21^. The polarization pattern generated by our converter is described in analogy to the spin Hall effect in a photonic cavity, making it an ideal system for application in spin-based devices that depend on the doping of a LC with an emitter. The matter component will lead to many spin textures with an anisotropic flow of particles^8,22^ carrying spin, such as spin whirls^15^, spin currents^12^, skyrmions^12,23^, half skyrmions^23^, hedgehog vortices^23,24^, hyperspin vortices^24^, pairs of half-vortices^25^ etc. We note the possibility of a device that would allow not only for manipulation of a light polarization pattern but also for a direct imprinting of light topology to a matter state in a single tunable converter.

## Methods

### Sample preparation

The sample consisted of two dielectric mirrors made of six pairs of SiO_2_/TiO_2_ dielectric layers, which were deposited on a 30 nm transparent electrode made of ITO grown directly on a quartz substrate. The top surface 60 nm of polyimide was spin-coated to ensure the homeotropic orientation of liquid crystal (LC) molecules. Mirrors were assembled to a cavity by thermopolymerizing glue with 0.9 μm glass spacers. The cell was filled with dual frequency LC (*n*_o_ = 1.504, *n*_e_ = 1.801) in a vacuum chamber by capillary action. Sample fabrication is discussed in detail in Supplemental information.

### Optical measurements

Optical measurements were performed at room temperature. For transmission measurements, two microscope objectives with numerical aperture NA = 0.55 were used. The size of the pump spot had a diameter of 1 μm. Based on the cavity Q-factor, we estimated the scattered photons propagation length as 7 μm. The transmitted laser wavelength was set to 1.599 eV. We could distinguish the polarization of the cavity modes using linearly polarized light of different orientations. To tune the cavity, we used an external voltage of square waveform of frequency 30 kHz.

### Simulations

Modeling was based on the so-called Berreman method^13^. In this approach, incident, transmitted and reflected electric fields in both TE and TM polarizations are connected by a 4 × 4 transfer matrix **T**. This method allows for calculation of the reflection and transmission coefficients of light incident on any system consisting of layers with given thicknesses and dielectric tensors on a TE-TM basis. We directly followed the formulation presented by Schubert^14^. Incident light with a given polarization state was first derived on a TE-TM basis for a given angle of incidence. Then, transmitted electric fields were calculated using transmission coefficients obtained directly from the **T** matrix (explicit formulas given in ref. ^26^). After changing the basis to the laboratory *x-y* coordinate system, the transmitted light intensities in *σ*^+^ and *σ*^−^ were calculated as $I_{I\sigma \pm } = \left| {E_x \pm iE_y} \right|^2$ and the degrees of circular polarization as

1 $$\rho _C = I_{\sigma + } - I_{\sigma - }/I_{\sigma + } + I_{\sigma - }$$

Calculations were performed for a structure consisting of two dielectric mirrors, each containing 5 pairs of layers with refractive indices *n*_high _= 2.2 and *n*_low_ = 1.45, with their thicknesses designed for a central wavelength of 700 nm. The two mirrors were separated by an anisotropic liquid crystal (LC) layer with dielectric tensor:

2 $$\hat \varepsilon _{{\rm LC}} = \left( {\begin{array}{*{20}{c}} {2.26202} & 0 & 0 \\ 0 & {2.26202} & 0 \\ 0 & 0 & {3.2436} \end{array}} \right)$$

which corresponds to ordinary and extraordinary refractive indices *n*_o_ = 1.504 and *n*_e_ = 1.801 and the molecular director initially oriented in the *z*-direction. The thickness of the liquid crystal layer was chosen to match the cavity mode energy at normal incidence as measured in a reflectivity measurement. The obtained thickness equal to 1.9 μm is close to the value estimated by the measurements :1.7 μm. Simulation of the voltage applied to the structure was introduced as a rotation of $\hat \varepsilon _{{\rm LC}}$ around the *y*-axis in the range of 0–30 degrees.

## Electronic supplementary material

Supplementary Information

## Data Availability

The datasets generated and analyzed during the current study are available from the corresponding author on reasonable request.
