# Supplementary material for: Tunable optical spin Hall effect in a liquid crystal microcavity
Source: Light Sci Appl. 2018 Oct 10;7:74. doi: 10.1038/s41377-018-0076-z (PMC6177461; doi:10.1038/s41377-018-0076-z)
Supplement: Supplementary file 1 — Supplementary Information [file 41377_2018_76_MOESM1_ESM.pdf]

Tunable optical spin Hall effect in a liquid crystal microcavity: supplementary information

Katarzyna Lekenta[1,*], Mateusz Król[1], Rafał Mirek[1], Karolina Łempicka[1], Daniel Stephan[1], Rafał Mazur[2], Przemysław Morawiak[2], Przemysław Kula[3], Wiktor Piecek[2], Pavlos G. Lagoudakis[4,5], Barbara Piętka[1], and Jacek Szczytko[1,**]

[1]Institute of Experimental Physics, Faculty of Physics, University of Warsaw, Poland
[2]Institute of Applied Physics, Military University of Technology, Warsaw, Poland
[3]Institute of Chemistry, Military University of Technology, Warsaw, Poland
[4]Department of Physics and Astronomy, University of Southampton, Southampton SO17 1BJ, UK
[5]Skolkovo Institute of Science and Technology Novaya St. ,100, Skolkovo 143025, Russian Federation
*katarzyna.lekenta@fuw.edu.pl
**jacek.szczytko@fuw.edu.pl

Substrates preparation

The microcavity with a liquid crystalline structure (LCMC) was assembled with using of a plane parallel plates made of a quartz glass (QP) 4 mm thick each. Both sides of QP were mechanically polished; the flatness of QP was better than λ/14 at λ = 633 nm, and wedge-shaped character of QP was smaller than 2 arcsec. The substrates were cleaned in a cascade washing system with ultrasonic agitation, using purified water of a specified resistivity r >18 MΩ×cm. After cleaning, the transparent conductive Indium Tin Oxide (ITO) electrodes (of thickness of $d_{ITO} = 30$ nm and refractive index $n_{ITO} = 1.890$ at λ = 632.8 nm) were deposited on the substrate. The ITO deposition process was carried out at substrates temperature of 200ºC under oxygen pressure of 7×10^{-5} mbar and at the XIAD ion gun assistance as to degas QP surfaces and for whipping the deposited layer. The Telemark 264 electron gun as a evaporation material source was used, quartz weight for controlling of evaporation rate and layer thickness, and heating system for temperature stabilization of substrates during processes.

Over the transparent, conductive ITO layer the distributed Bragg reflector (DBR) was made. DBR comprises of a 6 pairs of TiO_2 dielectric layers of a high refractive index ($n_{TiO2} = 2.436$ at $l = 0.6328$ µm, deposited at pressure 5×10^{-5} mbar of oxygen and temperature of 250°C at substrate) and low refractive index (SiO_2, $n_{SiO2} = 1.456$ at $l = 0.6328$ µm, 5×10^{-5} mbar oxygen pressure, 200°C substrates temperature). Vacuum evaporation process of electrodes (ITO) and dielectric mirrors were done using a XIAD ion source for whipping the deposited layers. The electrodes and mirror layer were shaped using of a special masks.

Filling the cavity with liquid crystal

As to prepare substrates for the aligning of the liquid crystalline structure, the ready QP substrates with transparent electrodes and DBR were spincoated (3500 rpm/25 sec) with SE 1211 polyimide (refractive index of $n_{PI} = 1.54$) solution (0.7% by wt. in dimethylacetamide - HPLC grade) and thermally conditioned as to polymerize the deposited prepolymer thin layer. The polyimide SE 1211 (by Nissan Chemicals Industries) was chosen as to ensure a normal (homeotropic) orientation of the molecular director of the liquid crystalline structure. Final polymer layer of thickness of c.a. 60 nm was a subject of a gentle rubbing as to avoid creation of domains while switching the LC structure from the homeotropic to homogenous one. Ready substrates were assembled in a form of a flat-parallel cell using a seal formed with using of a thermo-polymerizing glue with glass spacers of a nominal diameter of 0.9 µm. The seal was deposited in the vicinity of the substrate edges using an automatic fluid dispenser. The cell was placed in a special holder which was adopted for positioning of one substrate over the other. The holder allowed for tilting of the top surface. The parallel orientation of substrates was controlled by observation of interference fringes of a monochromatic light (yellow sodium

D lines) at normal incidence upon a gradual thermal polymerization of the seal. The cell gap was regarded flat-parallel while any traces of interference fringes disappeared. Finally, the cell was transferred to the vacuum chamber where the air was evacuated from the cell. In the vacuum chamber the cell's inlet was placed over the technological sponge immersed with LC. The cell was filled with LC while cell heating to the temperature of the isotropic phase of the LC. Finally, the cell was filled with LC by capillary actions.

Dual frequency LC

The nematogenic LC, dual frequency nematic mixture 1999C (DFNLC) was custom designed and synthesized at the Military University of Technology, Warsaw, Poland. This mixture exhibits a broad temperature range of the nematic phase existence and the cross-over frequency of order of 10 kHz at a room temperature (see Table S1). This mixture exhibits the change of the dielectric permittivity anisotropy $\Delta\varepsilon(f)$ value from positive (at low frequencies) to the negative one (at high frequencies) at the broad temperature range of the nematic phase[1] – see Fig. S1, Fig. S2 and Table S1.

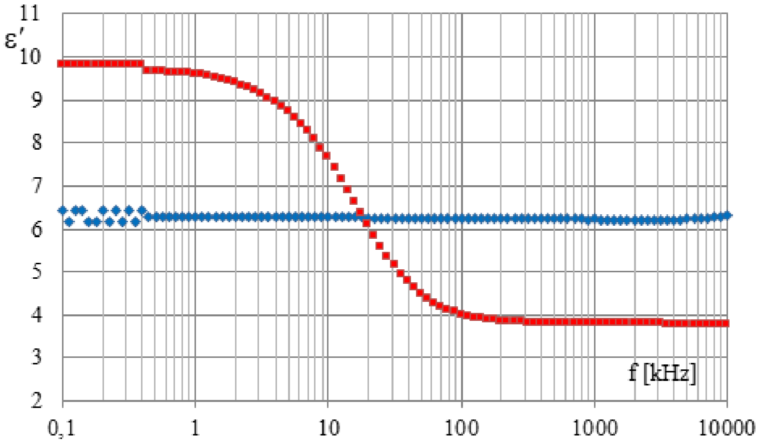

Fig. S1 The dispersion of the parallel (red squares) and perpendicular (blue rhombus) components of a real part ε' of the dielectric permittivity of the 1999C dual frequency nematogenic mixture at the temperature of 20°C. At the so called cross-over frequency f_C ~ 11 kHz is observed.

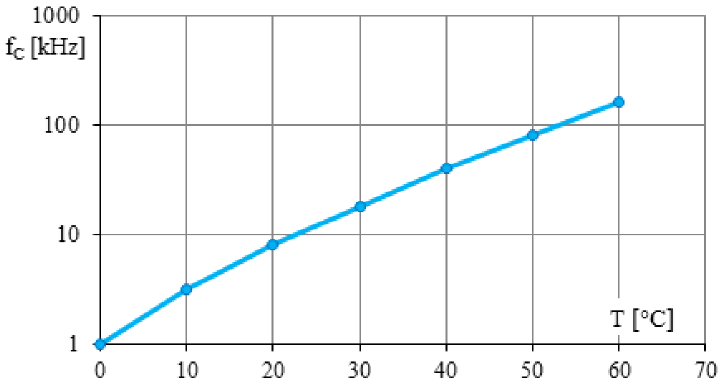

Fig. S2 The temperature dependence of the cross-over frequency of the DF nematic 1999C.

Tab. S1 Selected materials data of the DFNLC. T_{cr} – crystallization temperature obtained by DSC method (strongly depends on the thermal history of the sample), T_{Iso} – temperature of isotropization obtained DSC, Δn – refractive index anisotropy, f_c – cross-over frequency, $\Delta\varepsilon_{low}$ – dielectric permittivity anisotropy at low ($f << f_c$) frequency of the external electric field, $\Delta\varepsilon_{high}$ – dielectric permittivity anisotropy at high ($f >> f_c$) frequency of the external electric field.

Materials data of DFNLC (1999C)	
T_{cr} (°C)	<-20
T_{Iso} (°C)	146.4
Δn (589 nm) at 23°C	0.33
$\Delta\varepsilon_{low}$ (1 kHz) at 23°C	3.19
$\Delta\varepsilon_{high}$ (1 MHz) at 23°C	-2.91
f_c at 23°C. 1 kHz	8.35
f_c at 50°C. 1 kHz	79.4

Optical properties of DFNLC

The DFNLC in the cells gap at zero electric field ($U = 0$, $E = 0$), adopted the homeotropic orientation of the molecular director due to the DFNLC interaction with the cell's inner surfaces covered with SE1211 polyimide. For light passing through the cell at normal incidence, the DFNLC medium (at homeotropic orientation of the director) is perceived as optically medium of the effective refractive index n_{eff} ($U = 0$) equal the ordinary refractive index n_o of the DFNLC. At the alternating electric field E exceeding the threshold one E_{th} of frequency f significantly exceeding the cross over frequency fc, the molecular director tilts in plane of surface normal and rubbing direction. The effective refractive index n_{eff} for a light beam polarized in plane of rubbing direction (see Fig. S3) is given with equation (1). Since the effective refractive index can be tuned, the wavelength for the cavity mode can be affected with electric field.

$$n_{eff}(U) = \frac{n_o n_e}{\sqrt{n_o^2 cos^2(\Theta(U)) + n_e^2 sin^2(\Theta(U))}} \quad (1)$$

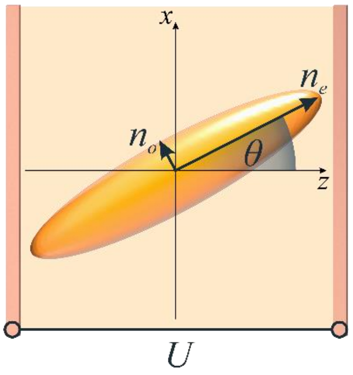

Fig. S3 The Optical indicatrix of the DFNLC while driving with electric field and the definition of parameters for the calculation of the effective refractive index n_{eff}.

Optical characterization of LCMC

In order to characterize obtained cavity modes tuning ranges angularly resolved spectra measurements were made for different voltages applied to the transducer electrodes. By applying the external electric field, we observe the H-polarized cavity mode shifting. Fig. S4 shows the photon mode tuning range for voltages varying from 0.0 to 9.5 V of square waveform of frequency 30 kHz. Thanks to the technology used in our device, we have the possibility of tuning the photon mode in a continuous way in a wide range of energy.

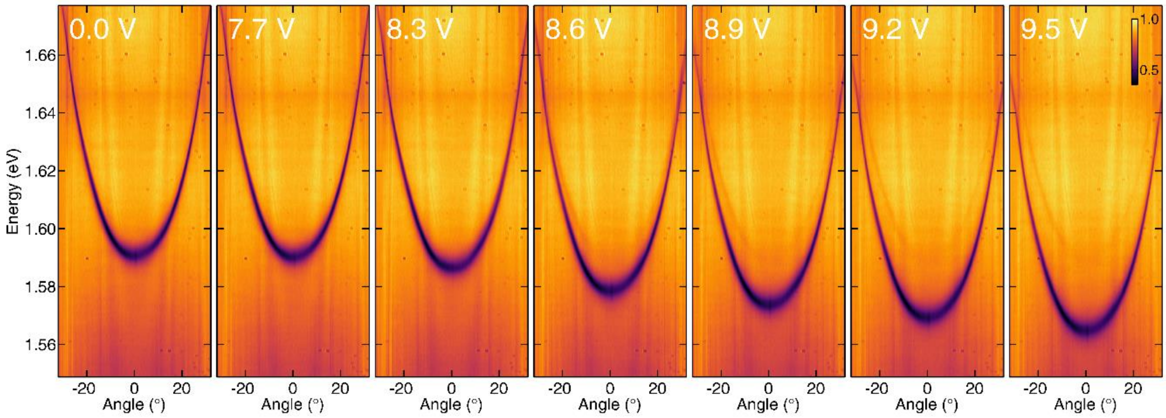

Fig. S4 Tuning of the H-polarized cavity mode. Applying a voltage causes rotation of the molecules, which leads to a change in the effective refractive index, thus to the tuning of the photon mode of the cavity. Angle-resolved reflectance spectra for voltages 0.0 V, 7.7 V, 8.3 V, 8.6 V, 8.9 V, 9.2 V and 9.5 V (panels from left to right).

[1] Mazur, R. et al. Nematic liquid crystal mixtures for 3D active glasses application. *Liq. Cryst.* **44**, 417–426 (2017).
